# Pressure Sensing in High-Refractive-Index Liquids Using Long-Period Gratings Nanocoated with Silicon Nitride

**DOI:** 10.3390/s101211301

**Published:** 2010-12-10

**Authors:** Mateusz Smietana, Wojtek J. Bock, Predrag Mikulic, Jiahua Chen

**Affiliations:** 1 Centre de Recherche en Photonique, Université du Québec en Outaouais, 101 rue Saint-Jean-Bosco, Gatineau, QC, J8X 3X7, Canada; 2 Institute of Microelectronics and Optoelectronics, Warsaw University of Technology, Koszykowa 75, 00-662 Warsaw, Poland; E-Mails: wojtek.bock@uqo.ca (W.J.B.); predrag.mikulic@uqo.ca (P.M.); jiahua.chen@uqo.ca (J.C.)

**Keywords:** optical fiber sensors, pressure sensor, long-period gratings, plasma deposition, thin films, silicon nitride

## Abstract

The paper presents a novel pressure sensor based on a silicon nitride (SiN_x_) nanocoated long-period grating (LPG). The high-temperature, radio-frequency plasma-enhanced chemical-vapor-deposited (RF PECVD) SiN_x_ nanocoating was applied to tune the sensitivity of the LPG to the external refractive index. The technique allows for deposition of good quality, hard and wear-resistant nanofilms as required for optical sensors. Thanks to the SiN_x_ nanocoating it is possible to overcome a limitation of working in the external-refractive-index range, which for a bare fiber cannot be close to that of the cladding. The nanocoated LPG-based sensing structure we developed is functional in high-refractive-index liquids (n_d_ > 1.46) such as oil or gasoline, with pressure sensitivity as high as when water is used as a working liquid. The nanocoating developed for this experiment not only has the highest refractive index ever achieved in LPGs (*n* > 2.2 at λ = 1,550 nm), but is also the thinnest (<100 nm) able to tune the external-refractive-index sensitivity of the gratings. To the best of our knowledge, this is the first time a nanocoating has been applied on LPGs that is able to simultaneously tune the refractive-index sensitivity and to enable measurements of other parameters.

## 1. Introduction

Long-period gratings (LPGs) have been known for over a decade [1]. LPGs are a periodic modulation of the refractive index along the length of the optical fiber. Under special phase-matching conditions, the grating couples the fundamental core mode to discrete cladding modes that are rapidly attenuated due to absorption and scattering. The coupling from the guided mode to cladding modes is wavelength-dependent, so one can obtain a spectrally selective loss. For the transmission spectrum of the LPG structure, two parameters can vary under the influence of an external stimulant: the resonance wavelength and the resonance transmission. The sensitivity is then typically defined as a shift of the resonance wavelength induced by a measurand [2]. A shift of resonance has been reported under a number of external influences including temperature, strain, bending and refractive index (RI) sensing e.g., [2]. Several applications of fiber grating structures in pressure sensing devices have been presented [3–5]. Recently we reported a very high pressure sensitivity of LPGs written in commercially available boron co-doped fiber [6]. The pressure sensitivity achieved for these gratings is at least 4 and as much as 8 times higher than for gratings written in other fibers which have been presented to date.

For most applications, pressure is generated using a liquid which is compressed and interacts hydrostatically with a sensing device. The sensing device should be then insensitive to the RI of the liquid and to its variations with pressure, thereby reducing the cross-sensitivity effect. Due to coupling of the cladding modes by the LPG structure, there is a significant dependence between the properties of the cladding, or its external media, and the spectral response of the LPG. The highest sensitivity of the LPG to the external RI can be observed when the external medium’s RI value is close to that of the cladding, which typically is made of fused silica (n_d_ = 1.458). In this particular case, the core of the fiber is surrounded by an infinite medium, and the cladding modes cannot be produced, so the coupling cannot take place [7]. Consequently, the resonances are not visible in the output spectra of the device, making the measurement impossible. The phenomenon has limited application of LPG-based sensing devices to those where water is used as the working liquid (n_d_ = 1.3334).

Reduction of the RI sensitivity has been already achieved by means of writing LPGs in a three-layered fiber [8] or recoating the LPG written in a standard fiber with a thick polymer overlay [9]. For pressure sensing none of these approaches is applicable. The commercially available three-layered fibers do not contain boron, which significantly reduces pressure sensitivity of the LPGs written in them. In the second case, coating the fiber with a thick polymer drastically changes spectral response of the LPG and makes it highly sensitive to variations in properties of the overlay versus a number of parameters. Moreover, a hysteresis in pressure response would appear in such a case, due to the relative softness of polymer materials, as well as due to the changes of the overlay’s optical properties in time, temperature and pressure.

In order to overcome problems mentioned above we applied a high-refractive-index (high-*n*) thin coating that can tune the intrinsic sensitivity of the LPG devices to a certain range of external RIs, while reducing the sensitivity in other ranges. A specially designed coating, in terms of its thickness and optical properties, optimizes the interactions of the light guided in the fiber and in the coating [10–13]. By modifying the guiding conditions of the cladding modes in this way, we can make the LPG insensitive to the RI of the liquid used in the pressure system. In order to fulfill durability requirements, the coating cannot change its properties under either long-term presence of the liquid or the applied pressure. The dense silicon nitride (SiN_x_) nanofilms deposited by the radio-frequency plasma-enhanced chemical-vapor-deposited (RF PECVD) method seem to be a perfect choice for this type of application. Due to their high hardness (∼19 GPa), excellent optical properties and very good adhesion to the silicon-related substrates, SiN_x_ films are typically applied as antireflective coatings for solar cells and as light-guiding layers in planar optical waveguide systems [14]. They exhibit high refractive index (*n* typically from 2 to 3.5) and negligible absorption in the infrared spectral range [15], both of which qualities are required in LPG-based sensors [16]. To the best of our knowledge, the nanocoating presented in this paper is the first ever applied to reduce the RI sensitivity of LPG-based pressure sensing devices.

## 2. Experimental Section

### 2.1. LPG Fabrication

In this experiment we used commercially available Fibercore PS 1250/1500 photosensitive fiber, where refractive indices of core and cladding defined at λ = 1,550 nm were 1.44937 and 1.4402, respectively. Gratings based on this fiber show very high pressure sensitivity [6]. Each of our samples contains a 10-cm section of the photosensitive fiber with the ends spliced to Corning SMF28 fibers. A set of LPGs was written with a computer-assisted precision arc-discharge apparatus, described in [4] and [17]. The LPGs were written only in the PS 1250/1500 fiber section. The discharge current was adjusted to be low enough to heat the fiber locally and not to produce any visible tapers due to the axial tension applied to the fiber. The arc discharge time was established at τ = 300 ms. The grating period was Λ = 375 μm and the length of the gratings was L = 20–25 mm. The optical transmission of the fiber in the range of λ = 1,160–1,660 nm was monitored during the LPG fabrication process in order to obtain the desired spectral attenuation notches. We used an Agilent 83437A broadband light source and an Agilent 86142B optical spectrum analyzer for this purpose.

### 2.2. SiN_x_ Nanofilm Deposition

The films were deposited using the Plasmalab 80+ system (Oxford Plasma Technology) working at f = 13.56 MHz [18]. SiN_x_ films were deposited on the LPG samples suspended 2.5 mm over the RF electrode, and simultaneously on wet oxidized silicon wafers to be used as reference samples which were placed directly on the electrode. The deposition procedure was similar to the one previously reported [19,20]. We employed a high [SiH_4_:N_2_]/[NH_3_] flow ratio (2% SiH_4_ diluted in N_2_) equal to 285/15 in order to obtain high-*n* SiN_x_ films. As for the other parameters for deposition, the RF power was 15 W, the pressure in the chamber was 0.53 mbar, the deposition time was from 3 to 8 minutes and the electrode temperature was 325 °C.

Film parameters such as the *n*, the extinction coefficient (*k*) and the thickness were determined by a Horiba Jobin-Yvon UVISEL spectroscopic ellipsometer on the silicon reference samples using a procedure described elsewhere [21]. Thickness of the SiO_2_ film deposited on the silicon wafers was measured to be in the range of 385 nm to 400 nm, which was thick enough to behave like the fused silica cladding of the fiber.

### 2.3. LPG Sensing Experiment

For pressure measurements, the LPGs were installed inside a steel housing in a transmission configuration [6]. To keep the fiber at a constant axial tension under different pressures, a fiber loop was formed inside the small inner channel of the housing, which was filled with Exxon instrument oil (n_d_ = 1.4793) and connected to a hydrostatic pressure standard DWT-35, capable of generating and calibrating pressures up to 100 MPa (accuracy of at least 0.1%). The pressure measurements were performed at room temperature (T = 23 °C). RI (n_d_) measurements of the liquids were performed using a VEE GEE PDX-95 refractometer working with an accuracy of ±10^−4^ refractive index units (RIU).

## 3. Results and Discussion

**Figure 1.**
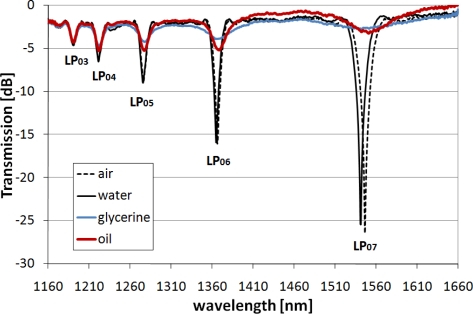
Influence of the external RI on the spectrum of LPGs (Λ = 375 μm) written in PS 1250/1500 fiber. RIs (n_d_) of water, glycerine and oil used as external medium are 1.3334, 1.4746 and 1.4793, respectively.

The aim of this work is to overcome the effect of the disappearance of the cladding modes when the RI of the surrounding liquid is close to that of the cladding, e.g., when an LPG is immersed in oils with a high RI. This situation is shown in Figure 1.

Since the higher-order cladding modes are more affected by the external RI when oil or glycerine is the surrounding medium, a spectral depth of several dB can be seen for the resonance caused by coupling of the higher-order modes (e.g., LP_06_ or LP_07_). Simultaneously, the pressure sensitivity increases with the order of the cladding modes [6]. Under such conditions, the measurement of pressure suffers from lack of precision in distinguishing the resonance minimum. The shift of such a shallow resonance is hardly traceable, in contrast to the resonance with a depth of 25 dB when water is used as the surrounding medium. Moreover, due to high-RI sensitivity, when the value of the external RI is close to that of the cladding, even small variations in the optical properties of the surrounding media will have a significant influence on the resonance wavelength [22]. This effect can be seen for both oil and glycerine, whose refractive indices are high and very close to each other. For such conditions, both effects—vanishing of the resonance and high RI sensitivity—disturb pressure measurements performed using an uncoated LPG-based device.

Both RI sensitivity and pressure sensitivity are known to increase with the order of the cladding modes [6]. To achieve coupling of high-order cladding modes, one should decrease the period of the grating to around 170 μm [2]. This can be done when the gratings are photo-induced. However, according to our previous experiments and to the results of others, UV photo-induced gratings in PS 1250/1500 fiber are not temperature-stable [23]. While RF plasma deposition can be performed in a relatively low-temperature process (even at room temperature [14]), the deposition temperature must be higher for SiN_x_ films [18] to ensure their quality, *i.e.*, to decrease their optical absorption, increase n and decrease roughness. However, degradation of LPGs takes place with less than 5 minutes of treatment at 350 °C, which is required for deposition of a good quality SiN_x_ film. As an alternative to UV writing, the LPG writing technique based on periodic electric-arc discharges [17,24] could be used. The arc technique, with its great simplicity, allows for flexible and low-cost LPG fabrication. The electric arc modifies the RI of the fiber core by releasing drawing-induced stress in the fiber. It has been shown that LPGs written in PS 1250/1500 fiber with the arc technique have a temperature-sensitivity similar to that of devices fabricated using UV irradiation [17]. However, the arc-written LPGs exhibit no change of the resonant transmission induced by thermal annealing during the deposition process, which makes them well suited for high-temperature deposition (350 °C) of nanocoatings [24].

### 3.1. Modification of Sensitivity to the External RI

**Figure 2.**
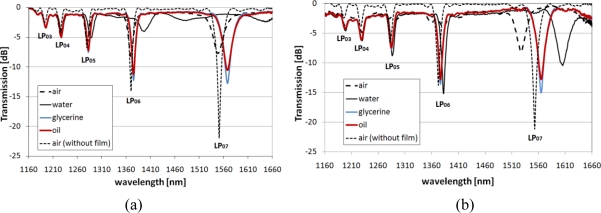
Effect of spectra recovery at high external RI for LPGs written in PS 1250/1500 fiber (Λ = 375 μm) coated with SiN_x_ film, where: **(a)** film thickness is 28.5 nm and *n* = 2.293 and **(b)** film thickness is 74 nm and *n* = 2.366. The spectrum measured for each grating before deposition of the film is given as a reference.

The effect of the deposition of two SiN_x_ films with different thicknesses and thus different *n* on the spectra of the arc-induced LPGs can be seen in Figure 2. The deposition times were 3 and 8 minutes. The properties of the films, as determined using reference samples, were: film thickness, 28.5 and 74 nm; and *n*, 2.293 and 2.366 (both at λ = 1,560 nm). The phenomenon of a lower *n* for SiN_x_ films thinner than 50 nm has been discussed elsewhere [25]. The *k* for both films were extremely low (close to 0) in the infrared spectral range.

Both spectra shown in Figure 2 experience a shift induced by deposition of the high-*n* nanocoatings, with a larger shift observed for the thicker and higher-*n* coating [Figure 2(b)]. Increasing the thickness or *n* of the coating can shift the maximum RI sensitivity to its lower values, while decreasing the sensitivity to the higher external RI [26,27]. We successfully shifted the maximum sensitivity of the LPGs from an external RI of 1.458 (for a grating with no film) to one in the range between 1 and 1.33. For both samples surrounded by any of the applied liquids, the coating is thick enough or has a high enough *n* to induce a transition of each of the cladding modes to lower-order ones. In this process, one of the modes begins to propagate in the coating, thus inducing the shift of other cladding modes to lower-order modes [28]. It can be seen from Figure 2(a) that for the LPGs with a thinner coating (28.5 nm), the high-order cladding modes are hardly guided when water surrounds the structure. When the coating is thicker (74 nm), the spectrum recovers well to below −10 dB for all of the applied liquids [Figure 2(b)]. With oil or glycerine surrounding the LPG, the spectrum shows a similar resonant wavelength to that of the uncoated sample surrounded by air. The results suggest that for these conditions the RI sensitivity is low, compared to that of the LPG with no coating when surrounded by the same oil or glycerine (Figure 1). However, due to the high extinction-coefficient of oil, the resonance is not as deep as for glycerine which has a lower extinction-coefficient and for which observed resonances are deeper for both investigated LPGs.

It must be mentioned here that there are several other nanocoating deposition techniques already applied to grow coatings on LPGs. However, the commonly applied deposition techniques show a series of disadvantages: they are time-consuming (e.g., electrostatic self-assembly), they are poorly controllable in terms of film thickness (e.g., sol-gel deposition) and in some cases the deposited films show low durability (e.g., Langmuir-Blodgett deposition). In order to maintain their long-term stability for such demanding applications as pressure sensors, the nanofilms must be very dense, a feature which is correlated to their high *n* and to their high optical quality. A higher *n* in the coating implies higher variations of the effective refractive index of the cladding modes as a function of the external RI and a higher shift in the attenuation bands [28,29]. That is why for the applications discussed here, the high-*n* coatings can be thinner and still have a comparable effect to thicker coatings with lower *n*. Moreover, it is known [16] that for some of the nanodeposition techniques mentioned above, the films suffer from a significant extinction-coefficient, which can cause the attenuation bands in the transmission spectrum to vanish. Conversely, plasma-deposited high-*n* SiN_x_ films show a very low *k* in the infrared spectral range [14] and seem to be a perfect choice for LPG-based pressure sensors capable of operating even in harsh environments.

### 3.2. Pressure Sensitivity of Nanocoated LPGs

As reported in our earlier paper [6], the resonances of LPGs fabricated in a boron co-doped fiber with a period of Λ = 375 μm experience a red shift. They reach linear sensitivities of 78, 56 and 45 pm/bar under the influence of pressure in a range of up to 240 bar, for the LP_07_, LP_06_ and LP_05_ cladding modes respectively. This is the highest pressure sensitivity reported so far for LPGs. The pressure sensitivity of the LPGs fabricated in the PS 1250/1500 optical fiber can be discussed in terms of the different elastic properties of the silica cladding and the B_2_O_3_/GeO_2_-containing silica glass core of the fiber [6]. The addition of B_2_O_3_ to SiO_2_ decreases the number of network bonds, and also decreases the bulk modulus (*K*) of the glass [30], *i.e.*, the resistance of the material to a uniform compression. The pressure-optic coefficient is dependent mainly on isothermal compressibility, that is the inverse of *K* [31]. The incorporation of B_2_O_3_ into fused silica in turn increases its pressure-optic coefficient, so that the core material has a higher pressure-optic coefficient than the cladding. When the influence of pressure on the period of the grating (Λ) is disregarded [6], Equation (1) shows a relation between the resonance wavelength ( $\lambda_{\text{res}}^{m}$) of an *m^th^* cladding mode, the effective refractive index of the propagating core mode ( $n_{\text{eff}}^{01}$) and the effective refractive index of the *m^th^* cladding mode ( $n_{\text{eff}}^{0m}$) induced by pressure (*P*) changes. The fact that the pressure-optic coefficient of the core is higher than that of the cladding explains the positive pressure sensitivity of LPGs written in the B_2_O_3_-containing fiber.

(1) $$\frac{\partial \lambda_{\text{res}}^{m}}{\partial P}=\Lambda \left(\frac{\partial n_{\text{eff}}^{01}}{\partial P}-\frac{\partial n_{\text{eff}}^{0m}}{\partial P}\right)$$

**Figure 3.**
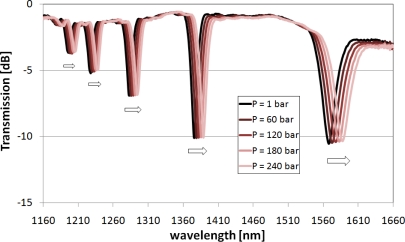
Spectral response of SiN_x_ coated (thickness 28.5 nm and *n* = 2.293) LPGs to pressure ranging from 1 to 240 bars. Arrows show the direction of the shift of the resonances induced by the increase of pressure. The measurements were performed in oil (n_d_ = 1.4793).

The response of the nanocoated LPGs to pressure changes is shown in Figure 3. Due to the thin (28.5 nm) high-*n* SiN_x_ coating, the resonances are clearly visible in the output spectrum of the grating, when the structure is operating immersed in a high-RI oil. The shift increases with the order of the cladding modes, just as was observed for the uncoated structure operating in water [6].

**Figure 4.**
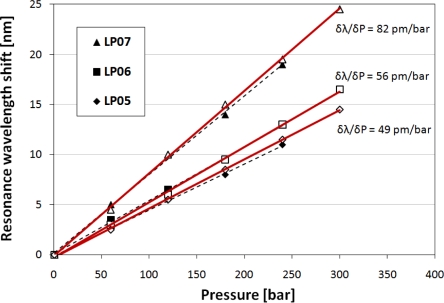
Pressure sensitivity for three observed resonances of the highest-order cladding modes comparing uncoated LPGs, investigated in water (black markers), and LPGs coated with SiN_x_ film (thickness of 28.5 nm and *n* = 2.293, white markers) investigated in high-RI oil.

When we compare the pressure sensitivity of both uncoated and coated LPGs, where the measurements were performed in water and oil, respectively, it can be seen that the results are in good agreement (Figure 4). A small difference for the LP_05_ and LP_07_ modes is most probably caused by a slight lack of repeatability of the grating fabrication process. It has been shown that even a very small tapering of the fiber during the arc-induced fabrication process can have a significant influence on the spectrum of the LPGs [17]. It is known that SiN_x_ can have a bulk modulus *K* more than five times higher than fused silica [32]. Following the relation between the pressure-optic coefficient and the bulk modulus of the material mentioned above, the pressure-induced change in the *n* of the SiN_x_ nanocoating is much smaller than the change experienced by either the cladding or the core materials. On the other hand, the nanometric thickness of the film makes it elastic and simultaneously does not disturb the pressure effect experienced by both core and cladding.

## 4. Conclusions

In this paper we introduce the idea of applying high-temperature plasma-deposited SiN_x_ nanocoatings to LPG-based pressure sensing structures. We show that due to the high *n* of this material (*n* > 2.2 at λ = 1,550 nm), even films only tens of nanometers thick can successfully tune the spectral responses of LPG pressure sensors. Furthermore, the SiN_x_ nanocoating overcomes the issue of the sensitivity of a bare LPG, which precludes its use in the external-RI range close to that of the cladding. We report a successful recovery of the grating spectrum for LPGs surrounded by high-RI liquid, and a simultaneous reduction of the RI sensitivity, both directly attributable to the application of the nanofilm. Taking into consideration the excellent adhesion of SiN_x_ film to silica surfaces, its high bulk modulus, its wear resistance and its low optical absorption, this coating can clearly be acknowledged as a good overlay material for robust LPG-based pressure sensing devices. The nanocoated LPG-based sensing structure presented in this paper works effectively even when surrounded by high-RI liquids such as oil or gasoline, with sensitivity as high as if water were used as the working liquid. The plasma-deposited SiN_x_ nanocoatings proposed here offer a good means of minimizing the cross-sensitivity of LPGs to other measurands, induced by RI sensitivity. The proposed application of thin and hard nanocoating to tune the refractive index sensitivity seems to be a good approach when, *i.e.*, polymer recoating is planned.
